# Targeting of Nrf2 improves antitumoral responses by human NK cells, TIL and CAR T cells during oxidative stress

**DOI:** 10.1136/jitc-2021-004458

**Published:** 2022-06-22

**Authors:** Stefanie Renken, Takahiro Nakajima, Isabelle Magalhaes, Jonas Mattsson, Andreas Lundqvist, Elias S J Arnér, Rolf Kiessling, Stina Linnea Wickström

**Affiliations:** 1 Department of Oncology-Pathology, Karolinska Institutet, Stockholm, Sweden; 2 Department of Clinical Immunology and Transfusion Medicine, Karolinska University Hospital, Stockholm, Sweden; 3 Gloria and Seymour Epstein Chair in Cell Therapy and Transplantation, Princess Margaret Hospital Cancer Centre, Toronto, Ontario, Canada; 4 Theme Cancer, Patient area Head and Neck, Lung and Skin, Karolinska University Hospital, Stockholm, Sweden; 5 Department of Medical Biochemistry and Biophysics, Karolinska Institutet, Stockholm, Sweden; 6 Department of Selenoprotein Research and National Tumor Biology Laboratory, National Institute of Oncology, Budapest, Hungary; 7 Clinical Neuroscience, Karolinska Institutet, Stockholm, Sweden

**Keywords:** immunotherapy, adoptive, lymphocytes, tumor-infiltrating, cytotoxicity, immunologic, T-lymphocytes, killer cells, natural

## Abstract

**Background:**

Adoptive cell therapy using cytotoxic lymphocytes is an efficient immunotherapy against solid and hematological cancers. However, elevated levels of reactive oxygen species (ROS) in the hostile tumor microenvironment can impair NK cell and T cell function. Auranofin, a gold (I)-containing phosphine compound, is a strong activator of the transcription factor Nrf2. Nrf2 controls a wide range of downstream targets important for the cells to obtain increased resistance to ROS. In this study, we present a strategy using auranofin to render human cytotoxic lymphocytes resistant toward oxidative stress.

**Methods:**

Melanoma patient-derived tumor infiltrating lymphocytes (TIL) and healthy donor-derived NK cells and CD19-directed CAR T cells were pretreated with a low dose of auranofin. Their resistance toward oxidative stress was assessed by measuring antitumoral responses (killing-assay, degranulation/CD107a, cytokine production) and intracellular ROS levels (flow cytometry) in conditions of oxidative stress. To confirm that the effects were Nrf2 dependent, the transcription level of Nrf2-driven target genes was analyzed by qPCR.

**Results:**

Pretreatment of human TIL and NK cells ex vivo with a low-dose auranofin significantly lowered their accumulation of intracellular ROS and preserved their antitumoral activity despite high H_2_O_2_ levels or monocyte-derived ROS. Furthermore, auranofin pretreatment of CD19 CAR-T cells or TIL increased their elimination of CD19 +tumor cells or autologous tumor spheroids, respectively, especially during ROS exposure. Analysis of Nrf2-driven target genes revealed that the increased resistance against ROS was Nrf2 dependent.

**Conclusion:**

These novel findings suggest that Nrf2 activation in human cytotoxic lymphocytes could be used to enhance the efficacy of adoptive cell therapy.

**Summary**

- Activation of antioxidant systems through Nrf2-activation improves lymphocyte resistance toward oxidative stress and thereby preserves their antitumoral efficiencies.

## Introduction

Adoptive cell therapy (ACT) using tumor infiltrating lymphocytes (TIL), natural killer (NK) cells or genetically modified NK-or T cells has proven very effective to treat patients with various advanced malignancies. Regardless of the promising results, there are still hurdles to overcome and room for improvement, which motivates the development of methods to enhance the ability of the infused T cells and NK cells to survive in the immune suppressive tumor microenvironment (TME) and eliminate cancer cells. Cancer cells and tumor-infiltrating immune cells, such as myeloid-derived suppressor cells, contribute to creating a hostile and immune suppressive TME, especially by the production of reactive oxygen species (ROS).^1 2^ ROS are chemically reactive oxygen derivates, such as superoxide radicals (O_2_^-^), hydroxyl radicals (OH) or hydrogen peroxide (H_2_O_2_). Physiological ROS play important roles as signaling molecules and reduction-oxidation (redox) processes regulate many biological processes, including immune responses.^3^ There are two major enzyme systems regulating the reductive pathways in cellular redox control, namely the glutathione (GSH) and thioredoxin (Trx) systems, driving a wide range of redox regulatory functions and antioxidant enzymes using reducing equivalents derived from NADPH (nicotinamide adenine dinucleotide phosphate).^4 5^

Controlled increased production of ROS is essential for innate immune cell functions against invading microbes and as antitumoral response.^3^ However, prolonged elevated ROS levels are also found during chronic inflammation and in cancer, and have been shown to diminish the immune response by inducing poor effector functions or cell death in T and NK cells.^2 6–8^ Early studies showed that ROS produced by autologous monocytes in the TME suppressed NK cell and T cell functions and their capability to response to cytokine activation.^9^ Other studies have shown that increased ROS levels leads to downregulation of the TCR/CD3 complex in T cells and the low-affinity Fc receptor FcγRIII (also known as CD16) on NK cells, resulting in reduced cytotoxic capacity.^10–12^ One possible way to target this deleterious effect on the immune system can be to lower the overall ROS levels in the TME.^13^ It has been shown that addition of histamine, Ceplene, can suppress ROS production by the monocytes and thereby preserve NK cell and T cell mediated tumor elimination in patients with acute myeloid leukemia.^14^

Another way to counteract the harmful effects of ROS on the immune system would be to activate the antioxidant systems within the cytotoxic lymphocytes, in order to increase their inherent resistance to oxidative stress in the TME. In this context Nrf2 (nuclear factor E2-related factor 2) is of interest as a transcription factor controlling a wide range of downstream targets that can help the cells obtain increased resistance to ROS accumulation. The activity of Nrf2 is regulated by constitutively expressed Keap1 (Kelch-like ECH-associated protein1), which in non-stressed cells binds to Nrf2 and promotes its degradation. Thioredoxin reductase 1 (TrxR1), a selenoprotein part of the thioredoxin systems, reduces oxidized cysteine residues on Keap1 and thereby regulates Keap1 function.^15^ During oxidative stress, or by inhibition of TrxR1, Keap1 is readily oxidized, allowing Nrf2 to enter the nucleus.^15–17^ Activated nuclear Nrf2 binds to antioxidant response elements initiating transcription of several detoxifying enzymes, thereby increasing the cellular resistance to higher levels of ROS.^15 18^ Pervious data shows that targeting Nrf2 by deletion or knockdown of Keap1 in human and murine T cells, respectively, renders the T cells more resistant toward oxidative stress.^19 20^ Furthermore, T cells from transgenic mice overexpressing thioredoxin (Trx1) were shown to be more resistant toward oxidative stress-mediated cell death and displayed improved antitumoral activities.^21^ Activation of the Trx system in tumor infiltrating NK cells also increased their antitumor activities after exposure to oxidative stress.^22^

Auranofin (AUF, Ridura) is a gold (I)-containing phosphine compound that was approved in 1985 to treat patients with rheumatoid arthritis, and is a very strong activator of Nrf2 as well as an inhibitor of TrxR1.^23–25^ Higher doses of AUF are cytotoxic and induce cell death in cancer cells and thus AUF is currently also being evaluated for anticancer therapy in clinical trials.^25–27^

In this study, we set out to investigate the possibility to establish a protocol for ACT resulting in an increased resistance toward oxidative stress and thus toward the higher levels of ROS present in the TME. We discovered that human cytotoxic lymphocytes indeed gain markedly increased resistance toward oxidative stress accompanied by an improved antitumoral efficacy on pretreatment with Nrf2-stimulatory and low non-toxic doses of AUF. Pretreatment of NK cells, TIL and CAR T cells with AUF resulted in clearly increased capacity of tumor elimination and cytokine release in the presence of H_2_O_2_ as well as during coculturing with ROS producing monocytes. We, thus, suggest that pharmacological activation of the intrinsic antioxidant pathways through Nrf2 activation could be a promising strategy to protect the effector functions of cytotoxic lymphocytes with strong antitumoral capacities. This principle can likely be used to improve the beneficial results of ACT.

## Materials and methods

### Cells

K562, RAJI, N6/ADR, THP-1, KADA and EBV-LCL feeder cells were cultured in RPMI with 10%–20% FBS (LifeTechnologies). KASUMI, ANRU and BEHA tumor cell lines in IMDM (LifeTechnologies) 20% FBS. Tumor cell line medium were supplemented with penicillin (100 U/mL) and streptomycin (100 µg/mL) (LifeTechnologies). Peripheral blood samples (anonymized blood donations from healthy adult donors) were purchased from Karolinska University Hospital Blood Bank. Peripheral blood mononuclear cells (PBMC) were isolated from healthy donor buffy coats using density centrifugation with Ficoll Paque Plus (GE Healthcare). NK cells and CD14^+^ monocytes were isolated from PBMCs using NK cell isolation kit or CD14^+^ microbeads, respectively (both Miltenyi Biotec), following the manufacturer’s instructions. ANRU monocytes were acquired through leukapheresis fraction 5. For additional details and description for melanoma patient derived TIL and CD19 directed CAR T cells, see online supplemental materials and methods.

10.1136/jitc-2021-004458.supp1 Supplementary data

### Treatment with compounds and oxidative stress

Lymphocytes were pretreated for 18 hours with Auranofin (AUF, with the concentration 1 µg/mL for NK cells and 0,5 µg/mL for TIL and CAR T cells, if not indicated differently), DL-sulforaphane (SUL) or dimethyl fumarate (DMF) (all Sigma-Aldrich) at indicated concentrations. As all compounds were reconstituted in DMSO, control NK cells were pretreated with DMSO with an equivalent volume to the highest compound concentration. 100 U/mL Catalase (Sigma-Aldrich) were added to control lymphocytes just before exposure to oxidative stress. For N-acetylcysteine (NAC, Invitrogen) treatment, DMSO pretreated cells were incubated for 1 hour with 5 or 10 mM NAC prior to H_2_O_2_ treatment. For Nrf2 inhibition, NK cells were pretreated with 50 µM ML-385 (Sigma-Aldrich) in parallel to AUF. For H_2_O_2_ treatment, lymphocytes were washed and resuspended to 1×10^6^ cells/mL in RPMI containing the indicated H_2_O_2_ (Sigma-Aldrich) concentration for 1 hour at 37°C 5% CO_2_. Cells were washed with medium and then used for further experiments.

### Coculture with autologous monocytes and detection of ROS produced by monocytes

See online supplemental materials and methods.

### Flow cytometry

All antibodies (see online supplemental table S1) and FACS reagents were used according to manufactures recommendation, if not stated otherwise. Cells were stained with LIVE/DEAD Fixable Aqua Dead Cell Stain Kit (Invitrogen) and then stained with the respective antibodies for 20 min at 4°C in PBS 1% FBS. Intracellular staining was performed using Fixation/Permeabilization Kit (BD Biosciences) following the manufacturer’s instructions. Samples without intracellular staining were fixed with 2% PFA (Thermo scientific) for 15 min before acquisition on a NovoCyte (ACEA Biosciences). All antibodies were titrated for optimal signal-to-noise ratio. Compensation was performed using AbC Total Antibody Compensation Bead Kit and ArC Amine Reactive Compensation Bead Kit (both Invitrogen). FlowJo Software (TreeStar) was used for analysis. For additional details regarding phenotypic analysis of NK cells (cell surface and intracellular markers), staining for intracellular ROS and flow cytometry analysis of spheroid-TIL cocultures, see online supplemental materials and methods.

### Assessment of effector functions

Lymphocytes were isolated and pretreated when indicated, with compounds and/or H_2_O_2_, as described above. For degranulation assay, NK cells were cocultured with K562 cells (E:T ratio 1:1) in the presence of anti-CD107a FITC antibody. After 2 hours, GolgiStop and GolgiPlug (BD Bioscience) were added and cells were harvested after an additional 4 hours coculture and stained for CD56, CD3 and anti-IFNγ, as described above. As a positive control, 25 ng/mL PMA (Sigma Aldrich) and 500 ng/mL Ionomycin (Sigma Aldrich) were added. For cytotoxic assay, a standard 4 hours (Cr51)-release assay was used. Briefly, target tumor cells were harvested and labeled with ^51^Cr (PerkinElmer). Effector cells, NK cells or T cel, were cocultured in 96-well V-bottom plate with labeled tumor cells at the stated effector to target ratio (E:T). The supernatant was transferred to LumaPlates (Perkin-Elmer) and radioactivity/tumor cell lysis was detected by MicroBeta2 (Perkin Elmer). Target cell killing was measured as % specific lysis and calculated using the formula: ((experimental release-spontaneous release)/(maximum release-spontaneous release))×100. For analyzing ADCC, NK cells were cultured with RAJI cells supplemented with rituximab (0,5 µg/mL, MabThera, Roche) or ofatumumab (0,05 µg/mL, Arzerra, Novartis). For cytokine release assay, NK cells and TIL were cultured with indicated tumor cells at the effector target ratio 4:1 (E:T) for 24 hours. IFNγ secretion was measured using human IFN-γ ELISA development kit (Mabtech) following the manufacturer’s instructions. For the 3D killing assay, spheroid (see above) and10^4^ AUF pretreated TIL (see above) were cocultured and apoptosis in the spheroid was monitored for 48 hours with the IncuCyte S3 live cell imaging system (Essen Bioscience, Sartorius) in the presence of CellEvent Caspase 3/7 Green detection agent (Invitrogen). Analysis was performed with the Incucyte software.

### Confocal microscopy

See online supplemental materials and methods.

### Evaluation of Nrf2 target gene expression

See online supplemental materials and methods.

### Quantification and statistical analysis

All statistical analyses were done using GraphPad Prism Software (V.9, San Diego, California USA). Used statistical tests as well as sample sizes are indicated in the figure legends. If not indicated differently, data are shown as mean±SD One data point (biological replicate) represents the mean of the respective technical replicates (n=3 for ^51^Cr release assay, n=2 for cytokine release assay, n=1 for flow cytometry). Statistical significance was defined as ***p<0.001, **p<0.01, *p<0.05. Due to variations between individuals, paired t-tests were used for NK and CAR T cell experiments. The sample size for TIL experiments was limited by availability of primary material.

### Additional resources

Confocal images were taken at Biomedicum Imaging Core, Karolinska Institutet. Graphics were created with BioRender.com.

## Results

### Targeting intrinsic antioxidant pathways in NK cells and TIL preserves their efficient antitumor responses after H_2_O_2_ exposure

**Figure 1:**
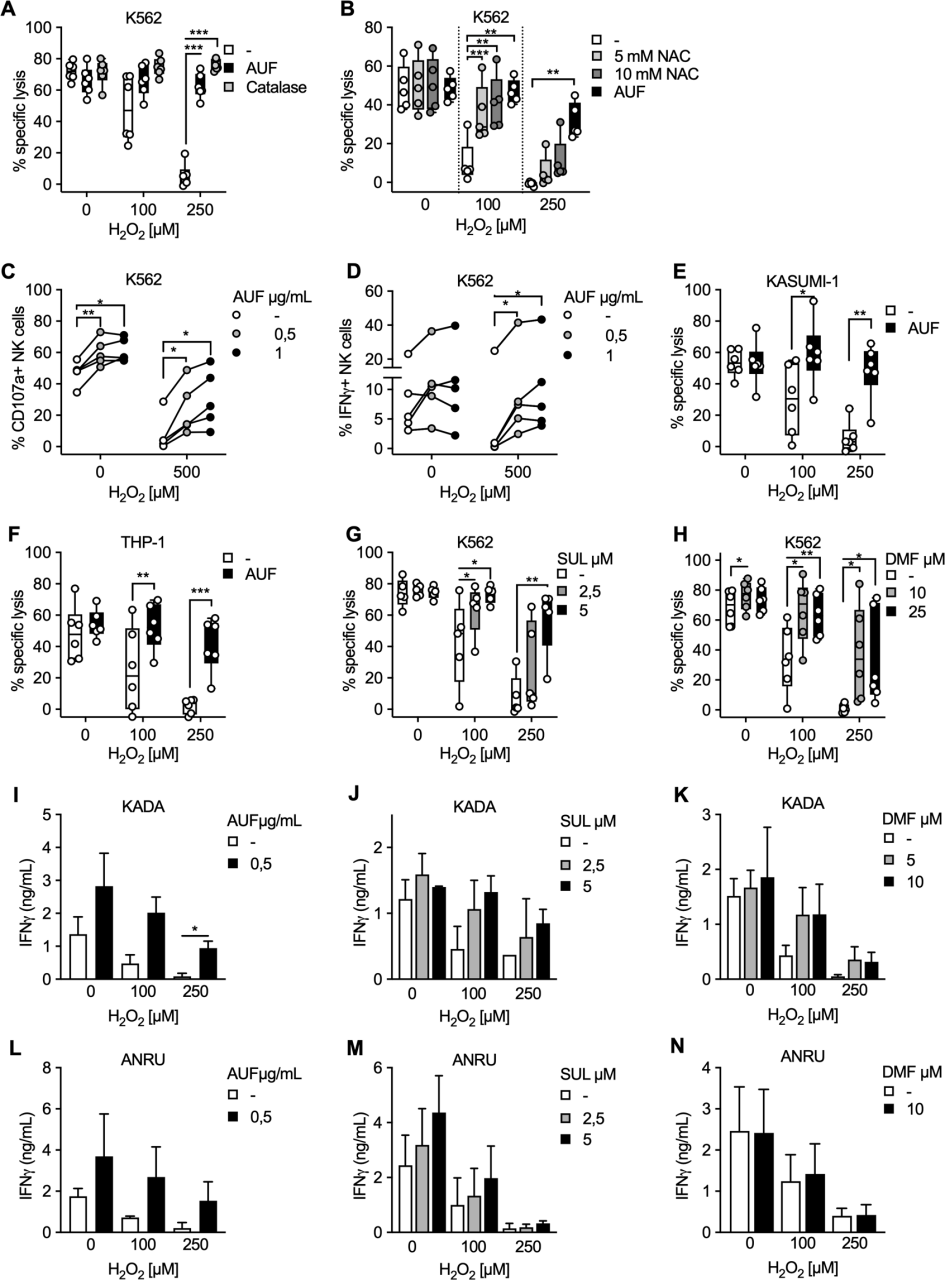
Targeting intrinsic antioxidant pathways in natural killer (NK) cells and tumor infiltrating lymphocyte (TIL) preserves their efficient anti-tumor responses after H_2_O_2_ exposure. NK cells or TIL were pretreated with indicated Nrf2 activating compound, exposed to indicated concentration of H_2_O_2_ and cocultured with indicated tumor target. Tumor target lysis is measured by 51Cr assay. (A) Lysis of K562 tumor cells by AUF pretreated or control NK cells. When indicated, catalase was added to the control samples just before exposure to H_2_O_2_, n=6. (B) Lysis of K562 cells by NK cells pretreated with AUF or DMSO and the control cells were analyzed with and without pretreated with NAC for 1 hour prior to the H_2_O_2_ treatment, n=5. (C–D) AUF pretreated and control NK cells were exposed to H_2_O_2_ at indicated concentration, cocultured with K562 tumor cells for 6 hours, and tumor recognition was measured by degranulation (CD107a expression) (C) or cytokine production (IFNγ expression) (D) by flow cytometry, n=5. (E–F) Lysis of KASUMI-1 (E) or THP-1 (F) tumor cells by AUF pretreated or control NK cells, n=6. E-F Lysis of K562 cells by control NK cells or NK cells pretreated with SUL (G, n=5) or DMF (H, n=6). (I–N) TIL from two melanoma patients, KADA (I–K) and ANRU (L–N), with or without pretreatment with AUF (I, n=2 and L, n=3), SUL (J, M, n=2) or DMF (K, N, n=2), were cocultured with their autologous tumor cell line. Tumor recognition measured by IFNγ release using ELISA. Statistical analysis: (A–H) paired t-test. (I–N) unpaired t-test. *P<0.05, **p<0.01, ***p<0.001. Each data point represents one NK cell donor (A–H). Effector: target ratio 9:1 (A–B, E–H), 1:1 (C–D) or 4:1 (I–N). Error bars in bar plots show mean with SD. Box plots show the median with error bars from minimum and maximum point. Auranofin (AUF), Sulforaphane (SUL), Dimethyl Fumarate (DMF), N-acetylcysteine (NAC), Dimethyl Sulfoxide (DMSO). Melanoma patients: KADA and ANRU.

Exposing expanded healthy human NK cells to H_2_O_2_ significantly decreases their ability to kill K562 target cells in a dose-dependent manner, as was expected (figure 1A, online supplemental figure S1A). However, notably this detrimental effect of H_2_O_2_ was almost completely counteracted by pretreatment of the NK cells with low AUF concentrations (figure 1A, online supplemental figure S1A). In addition, NK cells pretreated with AUF had overall improved viability following exposure to H_2_O_2_ (online supplemental figure S1B). Importantly, the addition of catalase to the control group (NK cells pretreated with DMSO), an enzyme efficiently converting H_2_O_2_ to H_2_O and O_2_, could analogously to AUF pretreatment improve the function of the control NK cells (figure 1A). Furthermore, pretreatment of control NK cells with N-acetylcysteine (NAC), a precursor to cysteine with a free thiol (SH) group increasing intracellular cysteine and glutathione levels providing several protective effects toward oxidative stress,^28^ resulted in a similar protection against exposure to 100 µM H_2_O_2_, but showed less protection against increased H_2_O_2_ concentrations compared with AUF (figure 1B). Thus, we concluded that AUF pretreatment of NK cells provides a strong protective effect against ROS (H_2_O_2_).

We next found that NK cells pretreated with AUF also displayed an increased capacity to trigger degranulation and cytokine production after coculture with K562 target cells, as measured by CD107a and IFNγ expression via flow cytometry (figure 1C, D and online supplemental figure S1C). Notably, for CD107a the protective effect was observed both in the presence or absence of H_2_O_2_ (figure 1B). Concordant with the killing of K562 target cells, NK cells pretreated with AUF displayed significantly increased killing capacity toward two other acute myeloid leukemia (AML) cell lines, KASUMI-1 (myeloblastic) and THP-1 (monocytic). These results support that AUF pretreatment of NK cells can intrinsically enhance NK cell degranulation, and result in a robust and efficient antitumoral capacity also in the presence of ROS and with several different tumor target cells (figure 1E, F).

In addition to AUF, other compounds such as SUL or DMF are known to activate Nrf2.^29^ We, therefore, investigated if SUL and DMF resulted in similar protective effects against ROS. NK cells pretreated with either SUL or DMF indeed resulted in comparable protective effects as AUF (figure 1G, H).

To further evaluate the effects of activating Nrf2 in cell products used for ACT, and to investigate if the effects could be extended to T cell products, we pretreated TIL from two melanoma patients, KADA and ANRU, with either AUF, SUL or DMF. Their resistance to H_2_O_2_ was subsequently investigated by measuring cytokine (IFNγ) production. TIL pretreated with any of the compounds resulted in increased recognition of their autologous tumor cells measured by IFNγ production, especially after exposure to H_2_O_2_, as compared with the control group (figure 1I–N). Comparably, specific lysis of the autologous tumor cells by KADA TIL was significantly increased on AUF pretreatment after exposure to ROS (online supplemental figure S1D). In concordance to NK cells, ANRU TIL pretreated with AUF displayed improved viability after exposure to H_2_O_2_ (online supplemental figure S1E). Thus, we conclude that both expanded NK cell and TIL products clearly display improved fitness after pretreatment with Nrf2 activating pharmacological compounds, especially when exposed to H_2_O_2_.

### Activation of typical Nrf2-dependent transcription patterns in human lymphocytes

**Figure 2:**
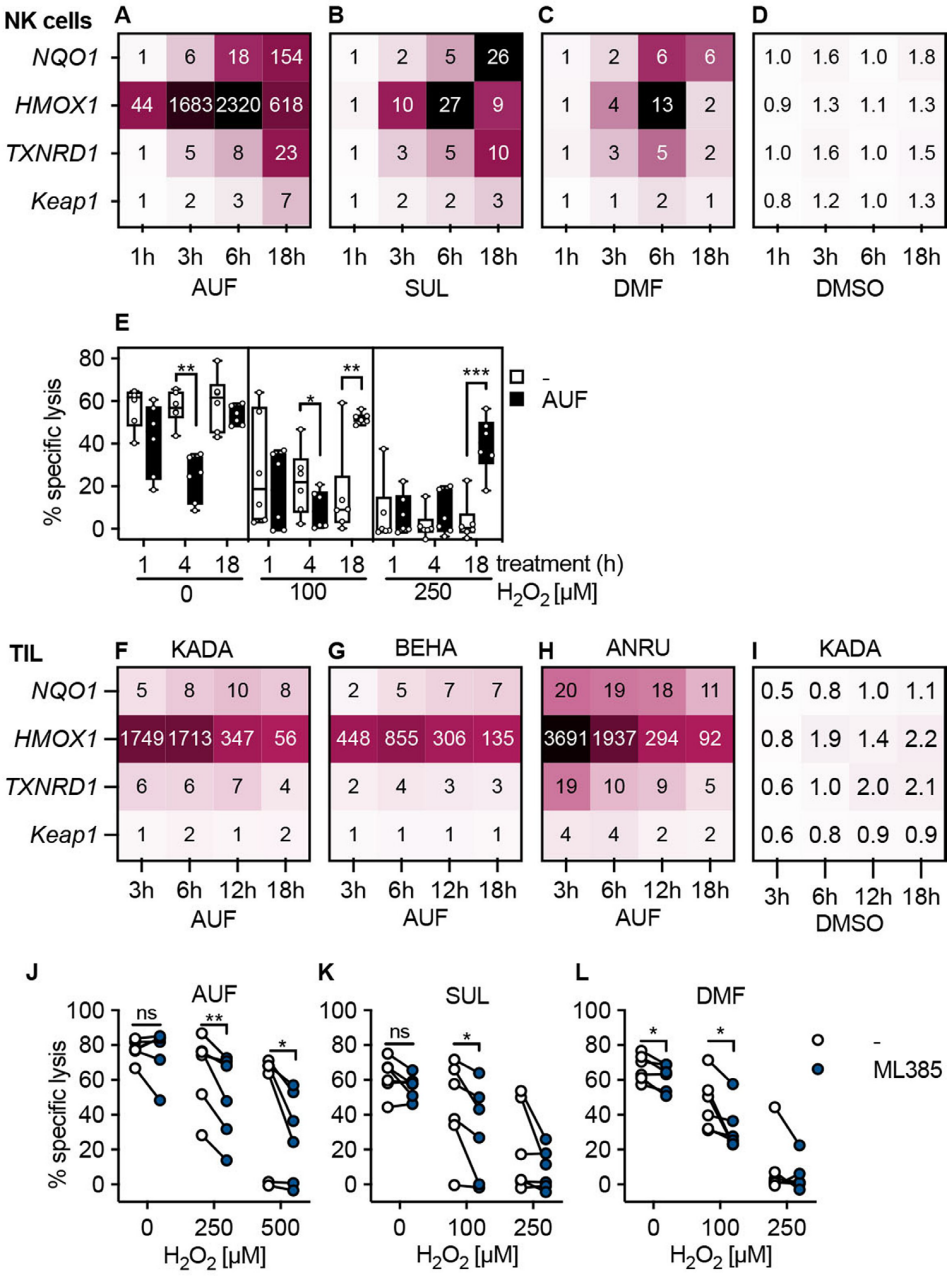
Activation of typical Nrf2-dependent transcription patterns in human lymphocytes. Validation of Nrf2 activation through qPCR of classical Nrf2 target genes or through Nrf2 inhibition in control versus compound treated lymphocytes. (A–D) Quantification of Nrf2 target gene expression in NK cells pretreatment with AUF (A), SUL (B), DMF (C) or DMSO (D) for indicated treatment duration using qPCR. Values represent the mean fold change gene expression (compared with untreated), n=4 (AUF), n=3 (DMF, SUL, DMSO). (E) Kinetic determining AUF pretreatment efficiency, measured by lysis of K562 tumor cells, E:T ratio 9:1, n=6. Box plots show the median with error bars from minimum and maximum point. (F–I) Quantification of Nrf2 target gene expression in TIL from three melanoma patients KADA (F), BEHA (G) and ANRU (H) pretreated with AUF or DMSO patient KADA (I). For DMSO controls for BEHA and ANRU see online supplemental figure S2C, D. (J–L) (F) KADA, (G) BEHA and (H) ANRU pretreatment with AUF. I–K Lysis of K562 tumor cells by NK cells pretreated with AUF (I), SUL (J), DMF (K) with or without the addition of the Nrf2 inhibitor ML385, n=6 donors. E, J–L Paired t-test, *P<0.05, **p<0.01, ***p<0.001. AUF, auranofin; TIL, tumor infiltrating lymphocyte. Auranofin (AUF), Sulforaphane (SUL), Dimethyl Fumarate (DMF), Dimethyl Sulfoxide (DMSO). Melanoma patients: KADA, BEHA and ANRU

Next, we wished to validate that the AUF, DMF and SUL pretreatment indeed activated the expected Nrf2-dependent antioxidant pathways in lymphocytes, as the increased expression of protective enzymes driven by Nrf2 could explain the increased resistance against H_2_O_2_. Therefore, we quantified the transcriptional levels of the classical Nrf2-targets *NQO1, HMOX1, TXNRD1,* as well as the Nrf2 inhibitor *Keap1,* by qPCR (figure 2A–D and F–H).^27 30^ Displaying the expression levels as fold changes compared with untreated NK cells or TIL, it was evident that for NK cells, AUF pretreatment resulted in a pronounced upregulation of *HMOX1* already at 1 hour; the expression of the other target genes more gradually increased over time with Keap1 being the least increased transcript (figure 2A). Sulforaphane and DMF had a comparable but less prominent influence on these Nrf2 target genes (figure 2B, C). No upregulation was seen in DMSO treated controls, showing that the observed effect was compound specific (figure 2D).

Based on the gene expression data, the effect of treatment time on protection of lymphocytic functional capacity was next investigated. We found that the beneficial effect and protection of their cytotoxic capacity occurred on 18 hours of AUF pretreatment for the NK cells (figure 2E). There was no difference comparing 18 hours and 24 hours pretreatment, or a 30 min AUF pulse followed by 17.5 hours incubation (online supplemental figure S2A, B). Auranofin pretreatment also induced robust activation of the Nrf2 target genes, most pronounced for HMOX1, in TIL from three melanoma patients, KADA, ANRU and BEHA (figure 2F–H). In line with the results obtained with NK cells, no activation of Nrf2-target genes was observed in DMSO treated controls (figure 2I, online supplemental figure S2C, D). However, the responses were faster than in NK cells, with the HMOX1showing the highest expression at 3 hours compared with the peak observed at 6 hours in NK cells. In agreement with this finding, a protective effect was observed in T cells already after 8–12 hours of AUF pretreatment (online supplemental figure S2C).

To confirm that the activation of Nrf2 is essential for the increased resistance toward H_2_O_2_ in cytotoxic lymphocytes, an Nrf2 inhibitor was used. For this, NK cells were pretreated with either AUF, SUL or DMF in combination with the Nrf2 inhibitor ML385,^31^ revealing that lysis of K562 was then significantly incapacitated on exposure to H_2_O_2_ (figure 2J–L). This strongly suggested that activation of Nrf2 indeed conferred the previously observed protective effects of AUF pretreatment. Notably, despite the addition of ML385 lysis was increased by NK cells pretreated with AUF as compared with the untreated controls (online supplemental figure S2D), which could either indicate additional mechanisms of action or an incomplete inhibition of Nrf2 activity. Importantly, no effects of ML385 alone were observed in control NK cells (online supplemental figure S2E).

Together, these findings demonstrate that activating the Nrf2 pathway can be used to improve lymphocyte’s resistance against oxidative stress and thus their ability to exert cytotoxic functions.

### Increased antitumor activities remain up to 72h after AUF pretreatment

**Figure 3:**
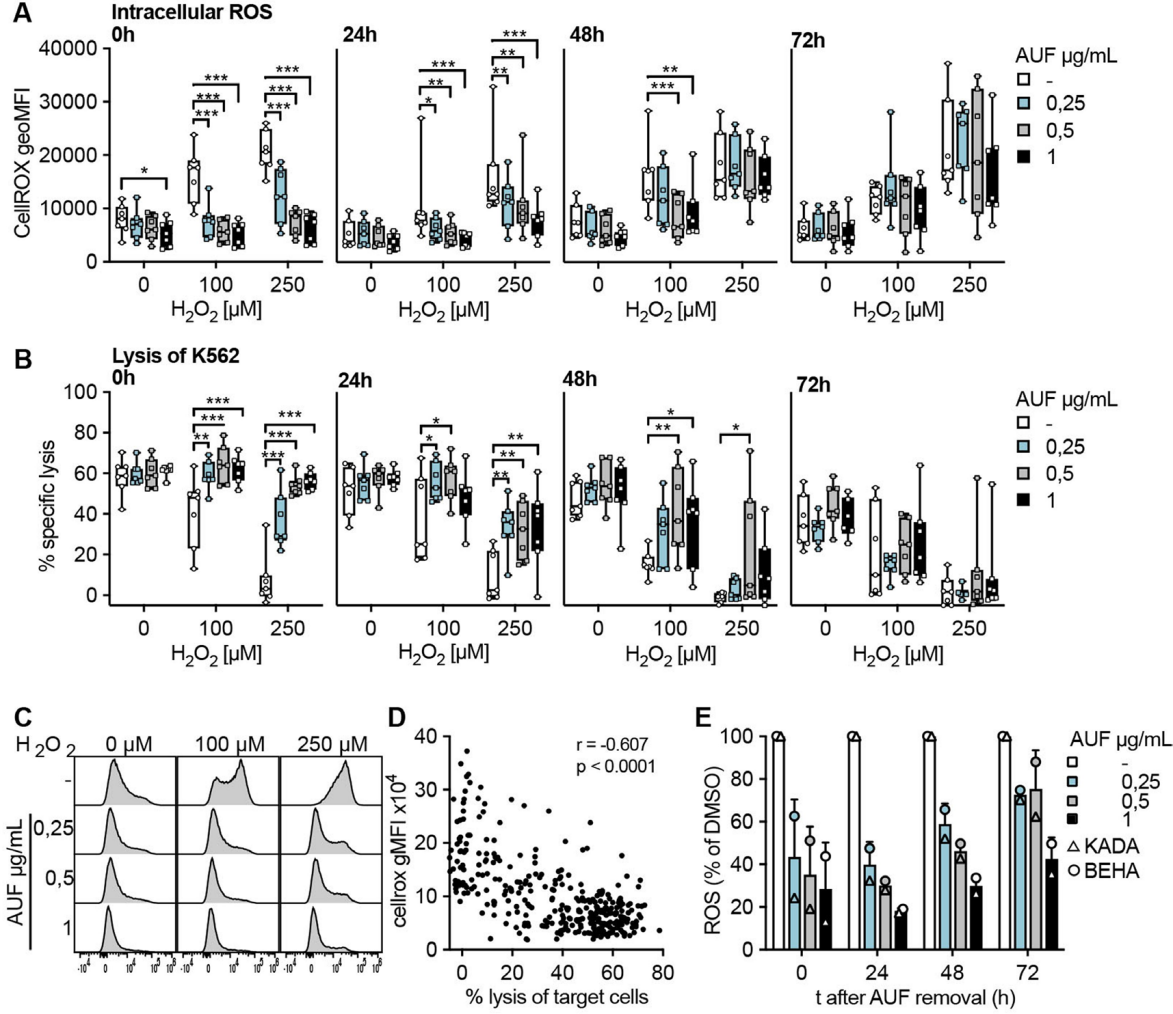
Increased antitumor activities remain up to 72 hours after auranofin (AUF) pretreatment. Natural killer (NK) cells or tumor infiltrating lymphocytes (TIL) were pretreated with auranofin and cultured without AUF for the indicated time (t;hours) before exposed to H_2_O_2_. For NK cells, intracellular reactive oxygen species (ROS) levels (A) and lysis of K562 tumor cells (E:T 3:1) (B) were measured in parallel at 0 hour, 24 hours, 48 hours or 72 hours after AUF removal, n=7. (C) Representative histograms show intracellular ROS levels (CellROX) in NK cells after ^H^_^2^_^O^_^2^_ treatment. (D) Spearman correlation between intracellular ROS levels and NK cell function with paired data points from A and B. (E) Intracellular ROS levels (CM-H2DCFDA) in TIL (n=2) 0–72 hours after AUF removal, calculated as relative to control. A-B 2-way ANOVA, *P<0.05, **p<0.01, ***p<0.001. Each data point represents one NK cell (A–B) or TIL donor (E). Bar plots show mean with SD. Box plots show the median with error bars from minimum and maximum point. Analysis of Variance (ANOVA).

For cells to be used in ACT it is important that any increased fitness would persist for a sufficient time following infusion into a patient, in order for the cells to be able to encounter and eliminate the tumor. Therefore, the kinetics of AUF pretreatment was investigated. For this, NK cells and TIL were pretreated with AUF and intracellular ROS levels as well as target cell lysis capacity, with or without addition of H_2_O_2_, were investigated up to 72 hours after AUF removal (figure 3). Intracellular ROS levels were assessed by flow cytometry using a cell-permeant dye that increases in fluorescence intensity on oxidation. In untreated NK cells, fluorescence was gradually increasing on exposure to increasing H_2_O_2_ concentrations (figure 3A, C), which also correlated with decreased function as seen by impaired target cell lysis (figure 3B, D). Of note, AUF pretreated NK cells displayed significantly lower intracellular ROS levels (figure 3A, C), which correlated with markedly improved effector functions up to 48 hours after AUF removal (figure 3A, B and D). Conversely, 72 hours after AUF removal the protective effects had diminished, as no differences in fluorescence or effector functions could then be detected between AUF pretreated NK cells and controls (figure 3A, B). Notably, even without the addition of H_2_O_2_, the AUF pretreatment resulted in significantly lower intracellular ROS levels (figure 3A). These results demonstrate that AUF treatment decreases intracellular ROS in NK cells.

In comparison to NK cells, intracellular ROS levels in the melanoma patient-derived TIL, KADA and BEHA, were in general higher (threefold) than in NK cells, why further increase by exogenous H_2_O_2_ treatment was difficult to detect by flow cytometry. Nevertheless, endogenous intracellular ROS levels were significantly lowered by the AUF pretreatment (figure 3E). In contrast to NK cells, this effect remained also at 72 hours after AUF removal. In line with the results obtained for NK cells, the effector functions of TIL pretreated with AUF displayed increased lysis capacity toward the autologous tumor cells. For the highest AUF concentration, this effect also lasted until 72 hours after AUF withdrawal (online supplemental figure S3).

These results collectively show that pretreatment with low non-toxic concentrations of AUF can improve the tolerance of NK cells and TIL toward oxidative stress, for as much as 2–3 days post-treatment, thus likely conferring an advantage for the cells to persist in an ROS-rich TME and promote tumor elimination.

### Auranofin pretreated NK cells display increased resistance against monocyte-derived ROS

**Figure 4:**
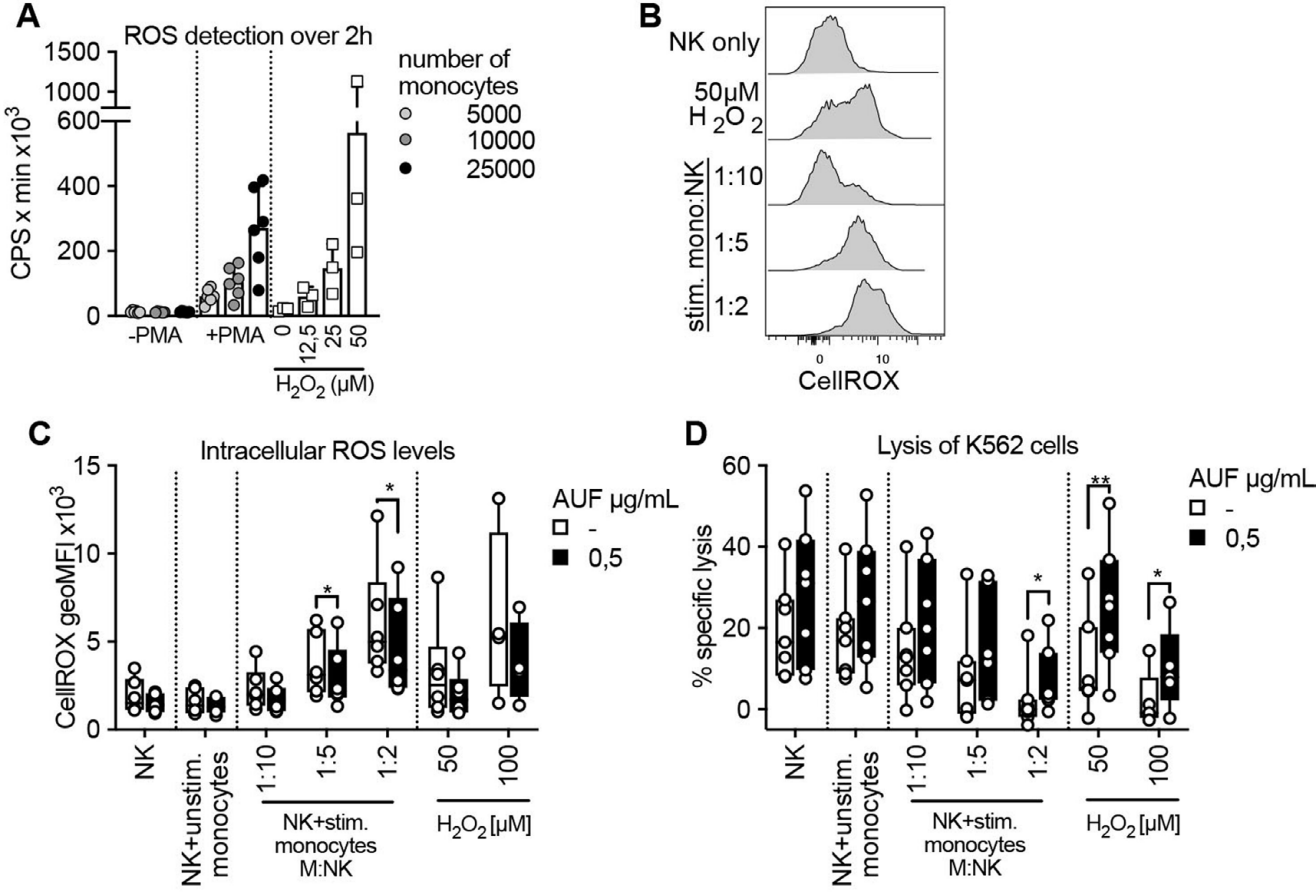
Auranofin (AUF) pretreated natural killer (NK) cells display increased resistance against monocyte-derived reactive oxygen species (ROS). AUF pretreated naïve healthy donor NK cells, with and without exposure to activated autologous monocytes, were analyzed for accumulation of intracellular ROS and lysis of K562 target cells. (A) ROS production by untreated or PMA activated monocytes detected by luminescence produced over 2 hours (counts per seconds, CPS), calculated as area under the curve, indicated H_2_O_2_ concentrations were used for reference, n=6. (B) Representative histograms show intracellular ROS levels (CellROX) in control NK cells after coculture with stimulated monocytes at indicated monocyte: NK cell ratio. (C–D) control or AUF pretreated NK cells were cocultured with autologous monocytes, with and without PMA preactivation, or exposed to indicated H_2_O_2_ concentration for 2 hourS and analyzed for (D) intracellular ROS levels and (E) lysis of K562 tumor cells (E:T 9:1), n=7. Unstimulated monocytes were used at 1:1 ratio. (D–E) paired t-test, *P<0.05, **p<0.01,. Each data point represents one NK cell donor. Bar plots show mean with SD. Box plots show the median with error bars from minimum and maximum point.

It has been shown that exposure to ROS, including H_2_O_2_, produced by autologous monocytes in the TME, leads to reduced NK-and T cell function.^1 2 9 32^ We, therefore, investigated if pretreatment with AUF could increase NK cell resistance toward ROS derived from activated monocytes, rather than exogenously added H_2_O_2_. To this end, healthy donor-derived monocytes were activated with PMA and their production of ROS was confirmed by a luminescence assay (figure 4A and online supplemental figure S4A). The ROS produced by activated monocytes in a 2-hour time course was compared with indicated H_2_O_2_ concentrations (figure 4A and online supplemental figure S4B). Coculturing such activated monocytes with naïve autologous NK cells, we next assessed the accumulation of intracellular ROS levels and the killing capacity of the NK cells toward K652 target cells. Increases in intracellular ROS in a dose (NK to monocyte ratio)-dependent manner could be observed using both untreated and AUF pretreated NK cells (figure 4B, C). However, the NK cells pretreated with AUF displayed significantly lower levels of intracellular ROS levels compared with control (figure 4C). Furthermore, the NK cells pretreated with AUF killed the K562 target cells with a markedly higher efficiency also in this setting, especially when exposed to the highest monocyte to NK cell ratio (figure 4D). Addition of catalase suppressed the differences between AUF pretreatment and controls, strongly indicating that the monocyte-derived suppression of NK cell function was to a major part H_2_O_2_ mediated (online supplemental figure S4C, D). This shows that AUF pretreatment of NK cells could be of relevance when ROS is produced by autologous immune suppressive cells.

### Auranofin pretreatment of NK cells and CAR T cells improve their antitumoral efficacies against hematological cancers

**Figure 5:**
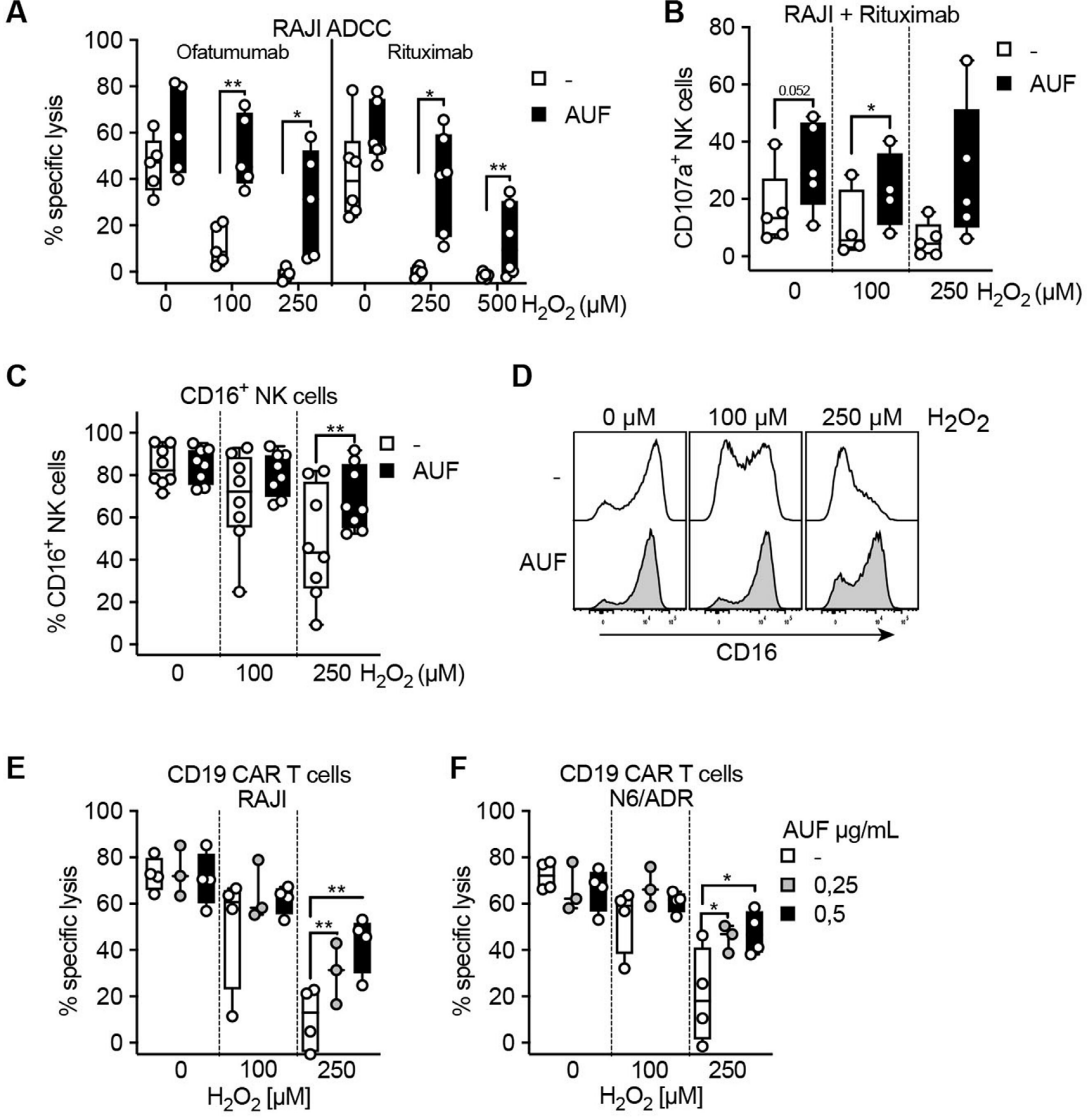
Auranofin (AUF) pretreatment of natural killer (NK) cells and CAR T cells improve their antitumoral efficacies against hematological cancers. NK cells and CD19 directed CAR T cells were pretreated with AUF, exposed to H_2_O_2_ and analyzed for lysis of CD19 and/or CD20 positive tumor cells, with or without the presence of anti-CD20 antibodies, or for CD16 expression. (A) AUF pretreated or control NK cell lysis of CD20+RAJI tumor cells in the presence of ofatumumab (n=5) or rituximab (n=6), measured by Cr^51^ release assay, E:T 9:1. (B) AUF pretreated or control NK cell recognition of CD20+RAJI tumor cells in the presence of rituximab measured by degranulation (CD107a expression), analyzed by flow cytometry, n=5. (C) Frequency of CD16^+^ NK cells 2 hours after H_2_O_2_ treatment measured by flow cytometry, n=8. (D) Representative histograms showing CD16 expression on control or AUF pretreated NK cells. (E–F) AUF or control CD19 CAR T cell lysis of RAJI (D) and N6/ADR (E) tumor cell lines, E:T 15:1, n=3–4. A, C–F paired t-test. Each data point represents one NK cell or CAR T cell donor. *P<0.05, **p<0.01,. Box plots show the median with error bars from minimum and maximum point.

To further investigate the potential of implementing AUF pretreatment in a clinical setting, we applied this pretreatment to ACT products used in the clinic. NK cells and CAR T cells have been demonstrated to show the highest efficiency against hematological cancer.^33 34^ Patients with B cell malignancies are today typically treated with therapies targeting CD20 or CD19, commonly using antibody therapies targeting CD20, Rituximab and Ofatumumab, or CD19 directed CAR T cells. To this end, we explored the effect of AUF pretreated NK cells in combination with anti-CD20 therapy. RAJI cells, a CD20^+^CD19^+^ lymphoma cell line (online supplemental figure S5A), were poorly recognized by NK cells (online supplemental figure S5B). However, when coated with either Rituximab or Ofatumumab, an efficient recognition of the target cells was observed, as detected by killing and degranulation (CD107a) assays (figure 5A, B). We found that NK cells pretreated with AUF displayed an increased recognition of RAJI cells, even without the addition of exogenous H_2_O_2_ (figure 5A, B). On addition of H_2_O_2_, we observed a significantly increased ability to lyse and degranulate in response to target cells by the AUF pretreated NK cells compared with controls (figure 5A, B). Phenotypic analysis of cell surface markers showed a significant decrease in the frequency of CD16^+^ NK cells after exposure to exogenous H_2_O_2_ (figure 5C and online supplemental figure S6), in line with previously published data.^10 12^ Compared with control cells, a significantly higher proportion of the NK cells pretreated with AUF remained CD16^+^ on exposure to H_2_O_2_ (figure 5C, D). As mentioned above, we detected that NK cells recognized RAJI cells weakly without addition of anti-CD20 (online supplemental figure S5B). This emphasizes the importance of combining antibody mediated immunotherapy with an approach that alleviates the detrimental effect of factors such as ROS in the TME.

**Figure 6:**
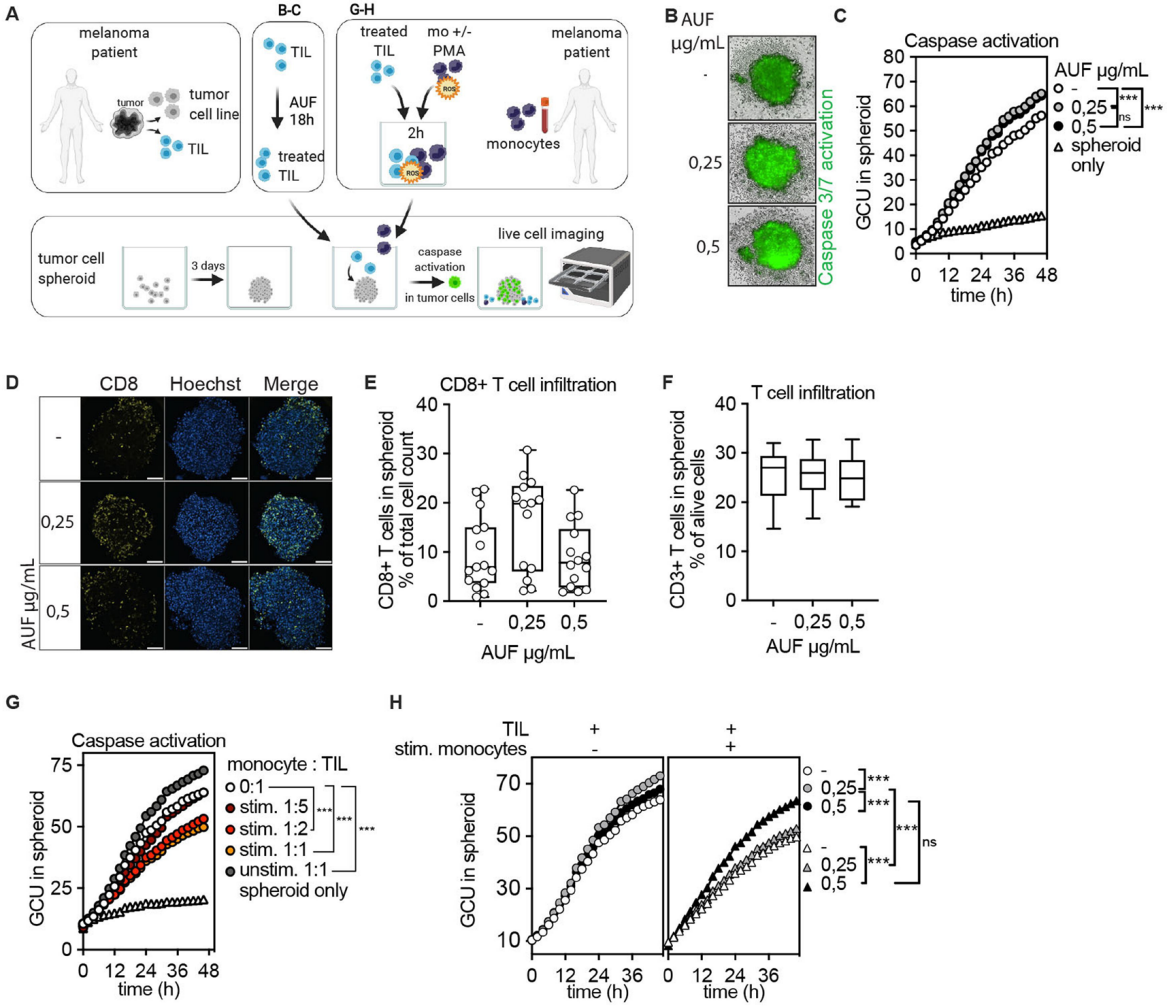
Increased induction of apoptosis in tumor spheroids by autologous tumor infiltrating lymphocytes (TIL) pretreated with auranofin (AUF). AUF pretreated ANRU TIL, with and without exposure to activated autologous monocytes, were analyzed for infiltration or induction of apoptosis in autologous tumor spheroids. Killing/Caspase activation was measured by green fluorescent intensity, GCU. A Cartoon displaying experimental setup. (B–C) AUF pretreated or control ANRU TIL killing of autologous tumor spheroids measured by caspase activation B) Representative images of autologous ANRU TIL-spheroid coculture displaying caspase activation, t=48 hours. (C) Quantification of caspase activation in spheroid boundary at indicated time of coculture. Graph shows the mean of four independent experiments. (D) ANRU CD8+T cell infiltration into autologous ANRU spheroid at 6 hours of coculture, analyzed by confocal microscopy where CD8+T cells are shown in yellow and nuclei in blue. Scale bars 100 µm. (E–F) Quantification of CD8+ (E) or CD3+ (F) ANRU TIL infiltration into ANRU spheroids using confocal imaging (each data point represents one spheroid, two independent experiments) or flow cytometry (n=6), respectively. (G) ANRU TIL mediated autologous tumor spheroid killing with and without presence of activated autologous monocytes, measured by caspase activation. Graph shows the mean of 2 independent experiments. (H) AUF pretreated or control ANRU TIL mediated autologous tumor spheroid killing with and without presence of activated autologous monocytes (1:1 ratio), measured by caspase activation. C, F, K one-way ANOVA with Turkey’s multiple comparisons test. ***p<0.001. Box plots show the median and extend from minimum to maximum point.Analysis of Variance (ANOVA).

We next investigated if AUF pretreatment could improve the effects of CD19 directed CAR T cells in an ROS-rich environment. A significant increased killing of CD19 +lymphoma and leukemia target cells (RAJI and N6/ADR, respectively, online supplemental figure S5A) by CAR T cells pretreated with AUF as compared with controls was observed (figure 5E, F). Thus, AUF pretreatment can potentially improve already approved cancer therapies, such as antibody therapies or CAR T cell-based ACT.

### Increased induction of apoptosis in tumor spheroids by autologous TIL pretreated with AUF

To model targeting of solid tumors, we next investigated if AUF pretreatment of TIL could increase their capacity to eliminate autologous tumor cells grown as spheroids (figure 6A). For this, untreated or AUF pretreated ANRU TIL were cocultured with autologous tumor spheroids. A fluorescent green dye quantifying apoptosis via Caspase 3/7 activation was used to measure tumor elimination. ANRU TIL pretreated with AUF displayed a substantially increased capacity to induce apoptosis in the tumor cells as compared with control cells, as shown by the increased green fluorescent intensity indicating caspase activation (figure 6B, C). Notably, there was no difference in the capacity of the TIL to infiltrate the spheroid, as assessed by flow cytometry and confocal microscopy (figure 6D, F). Thus, the increased capacity of TIL pretreated with AUF to kill the tumor cells was not due to an increased infiltration, but rather due to improved effector functions. To further enhance the levels of oxidative stress in this model, untreated ANRU TIL were exposed to ROS as released by activated autologous monocytes at different ratios (figure 6G and online supplemental figure S7A). TIL cocultured with non-activated monocytes (unstim 1:1) had an increased capacity to eliminate the tumor spheroids as compared with TIL cultured without addition of monocytes (0:1) (figure 6G and online supplemental figure S7B). This may be expected, since monocytes are capable of stimulating T cell activation.^35^ However, increasing the number of activated monocytes per TIL caused an evident decrease in TIL capacity to induce apoptosis in the tumor cells grown as spheroids. An increased killing capacity was observed in spheroids cocultured with TIL pretreated with the highest dose of AUF also in this setting (figure 6H). In addition, monocyte-derived ROS impaired cytokine release and lysis of two-dimensional-grown tumor cells by untreated TIL, which could also be partially rescued by AUF pretreatment (online supplemental figure S7C and data not shown).

We conclude that pretreatment of TIL with a compound activating Nrf2 significantly increases their resistance against monocyte derived oxidative stress and therefore improves their antitumoral activity. In addition, AUF pretreated TIL had a significantly increased capacity to induce caspase dependent cell death of tumor spheroids. This suggests a great potential for AUF pretreatment as an option of cell products used for ACT to treat solid tumors.

## Discussion

In this study, we show that compound-mediated activation of Nrf2 in long-term expanded human cytotoxic lymphocytes can be used to improve their fitness and tumor cell killing capacities. We suggest that repurposing of the FDA approved compound auranofin can thus be used to pretreat NK-, TIL and CAR T cell-products for ACT. Our results show that such pretreatment improves their tolerance to ROS and thereby enhance the antitumoral response on exposure to oxidative stress in the TME.

Auranofin and DMF are FDA-approved compounds used to treat patients with rheumatoid arthritis or multiple sclerosis, respectively.^24 36^ However, the three Nrf2 activating compounds, AUF, SUL and DMF studied herein are also extensively evaluated for their capacity to induce cell death for potential use in anticancer therapies.^15^ Furthermore, AUF has been shown to induce cell death in HIV-infected T cells.^37^ Thus, we conclude that the findings presented here provide another unexpected application for these three Nrf2 activating compounds, when used at lower non-toxic concentrations.

Noel *et al* showed that indirectly activating Nrf2 by deleting or knocking down Keap1 in human and murine T cells, respectively, can also be used to trigger an increased expression of classical Nrf2 target genes.^19 20^ However, CRISPR/cas9-mediated deletion of KEAP1 in human T cells seemed to preferentially work in CD4^+^ T cells, and increased the frequency CD4^+^cells with a regulatory phenotype being less favorable for anti-cancer immunotherapy.^19^ Notably, we did not observe any increase in frequency of CD4+T cells after AUF pretreatment and no induction of regulatory T cells could be detected at 0 hour or 72 hours post AUF pretreatment of KADA TIL (online supplemental figure S8). In addition, the different outcome may potentially be due to differences in the regulatory signaling pathways that may be triggered in genetic versus compound-mediated activation of Nrf2. Last but not least, genomic damage in mitotically active cells caused by CRISPR-Cas9 editing may potentially have pathogenic consequences,^38^ and treatment of lymphocytes for clinical use with an FDA approved compound will facilitate regulatory approval.

We found that the effect of AUF pretreatment on expanded NK cells and TIL lasted up to 48–72 hours, respectively. We previously showed in mice that a robust NK cell-mediated tumor killing in vivo occurs within 24 hours after injection and can be initiated already after 6 hours and data not shown.^39^ Similar to NK cells, there are reasons to believe that TIL mediated tumor elimination is initiated shortly after infusion. As early as 24 hours after injection of 111In-labeled TIL, localization of TIL to sites of metastatic deposits was demonstrated.^40^ In our clinical trial (NCT01946373), we witnessed one patient who responded within hours after the infusion and developed influenza-like-symptoms without IL-2 treatment,^41^ and a very rapid antitumoral response has been observed in CAR T cell treated patients.^42^ We thus think that the 48–72 hour ‘window’ of significantly lower accumulation of intracellular ROS and increased antitumoral efficacy of AUF pretreated cells as found here, should be long enough to ‘kick-start’ efficient tumor elimination in the patient. This could lead to increased antigen spreading and thereby also reactivation of non-infused T cells already present in the TME. Noteworthy, arguments for the importance of a rapid ‘hit-and-run’ mechanism of TIL cells is not at odds with the importance for the injected TIL to survive for a long time in the patient, even if the effect of the AUF then has decreased. One could discuss if systemic/in vivo administration of AUF could be an option potentially result in longer protection of NK cells/TIL against oxidative stress. However, AUF is being investigated for anticancer therapy due to its toxicity at higher concentrations, its FDA approved as a therapy for RA and known to have immunosuppressive effects.^43 44^ The novelty of our study, to protect cytotoxic lymphocytes used for ACT, is based on careful titration of the auranofin dose inducing the protective effect without high toxicity. The exact concentration, the protective ‘window’ will be difficult to achieve with in vivo administration and therefore less appealing for this purpose.

Alternative ways to increase resistance toward oxidative stress is by genetic modifications of other redox-related genes than Nrf2. Chakraborty *et al* showed that overexpression of Trx-1, in murine and human T cells, increased their resistance toward oxidative stress as observed by increased viability, altered glucose metabolism and preserved antitumor effector functions.^21^ Introduction of antioxidant enzymes, catalase and/or superoxide dismutase, has also been shown to protect several organs from oxidative stress mediated injury.^45 46^ Overexpression of catalase in human T cells preserved anti-viral recognition during H_2_O_2_ or granulocyte produced ROS.^47^ Furthermore, coexpression of catalase and a chimeric antigen receptor in human T cells resulted in improved antitumoral capacity during oxidative stress.^48^ Overexpression of catalase in CAR T cells reduced activation mediated intracellular ROS. Concordant with these other findings, we here observed that AUF pretreatment of CD19 directed CAR T cells had a protective effect against oxidative stress and therefore efficient killing of lymphoma and leukemia tumor cells was maintained (figure 5E–F). We have also observed lower intracellular ROS levels in AUF pretreated NK cells and TIL (figure 3A, E) resulting in significantly increased effector functions without exposure to oxidative stress (figure 1C, H). This suggests that AUF pretreatment can improve the function of NK and TIL cells even in the absence of additionally increased oxidative stress, thereby increasing the application scope of such AUF pretreated cell products to be used for ACT.

ACT aiming to eliminate solid tumors is today focused on the usage of TIL. In this study, we found that AUF pretreatment can protect melanoma-derived TIL from the detrimental effects of ROS, either added exogenously in the form of H_2_O_2_ or, more physiologically relevant, as produced by activated autologous monocytes. In addition, AUF pretreatment increased their capability to eliminate tumor cells grown as spheroids. Furthermore, AUF pretreated TIL had an improved capacity to induce caspase-dependent apoptosis in such spheroids, even without exogenous addition of H_2_O_2_.

For NK cells, it has been shown that CD56 ^bright^, immunomodulating and cytokine producing NK cells are more resistant to granulocyte produced ROS, as compared with cytotoxic CD56^dim^ NK cells.^8^ The authors conclude that the effect is H_2_O_2_ dependent since the CD56^dim^ NK cells could be rescued by the addition of exogenous catalase. In concordance with these findings, our expanded NK cell product mainly consists of cytotoxic CD56^dim^ NK cells and can be as efficiently rescued from H_2_O_2_ damaging effects by AUF pretreatment as by addition of exogenous catalase.

Previous studies have shown that oxidative stress can cause downregulation of TCR/CD3ζ and CD16/CD3ζ complexes on naïve T and NK-cells, respectively. This has been suggested a possible mechanism for how oxidative stress leads to impaired NK cell and T cell function.^10–12^ We found that pretreatment of NK cells with AUF could reduce ROS-mediated loss of CD16^+^ on NK cells. This motivated us to interrogate if AUF pretreatment of NK cells could improve anti-CD20 antibody therapies, since the clinical efficacy of antibody treatment targeting anti-CD20 treatments is partly mediated by NK cells. The Fc-region of the anti-CD20 antibody binds to the low affinity Fcγ receptor (FcγRΙΙΙ) CD16 on NK cells and thereby induces elimination of the B cells via NK cell mediated antibody-dependent cellular cytotoxicity (ADCC).^49^ We observed increased beneficial effect of AUF pretreatment of NK cells in the absence of oxidative stress and a significant increase after exposure to H_2_O_2_. This could be of considerable importance in relation to clinical outcome in NK cell ACT, since hematological cancers are known to harbor increased ROS levels.^50^ Additionally, combination of NK cell ACT and antibody therapy has proven safe, and resulted in reduced regulatory T cells in the blood as well as preliminary antitumor reactivity in patients with gastric and colon cancer.^51^ Thus, AUF pretreated NK cells combined with any antibody therapy inducing ADCC could be an attractive option to combat the negative influence of oxidative stress on the clinical outcome.

Although it is difficult to assess what a physiological dose of H_2_O_2_ corresponds to, we consider the doses of H_2_O_2_ used in this study to be physiologically relevant. This is supported by our observation that pretreatment of NK cells and TIL with AUF protected against autologous monocyte-derived ROS to the same extent as toward the H_2_O_2_ concentrations we added exogenously. Also, we used concentrations of H_2_O_2_ and monocyte numbers that gave similar signals of fluorescence with our ROS-detecting probes. It shall be noted, however, that activated monocytes or tumor cells can produce several types of oxidants, including H_2_O_2_, superoxide, lipid peroxides and more.^3^ These molecular species in combination may be more detrimental for T cell and NK cells than the effects observed after solely adding exogenous H_2_O_2_ at different concentrations. It was therefore important for us to also note the evident resistance of the cells pretreated with AUF also toward the oxidants produced by activated monocytes and tumor target cells.

Although Nrf2 activation mainly is regarded as a factor protecting us from oxidative damage, a negative side of Nrf2 activation in cancer is the enhanced resistance of cancer cells to oxidative stress and to chemotherapeutic agents including cisplatin and doxorubicin.^18 52^ Notably, the enhanced resistance to chemotherapeutic agents in AUF pretreated T cells or NK cells may instead be to the patient’s advantage, allowing ACT to be carried out simultaneously or soon after chemotherapy treatment.

Taken together, we have here shown for the first time that repurposing of an FDA approved compound targeting the intrinsic antioxidant pathways via Nrf2 activation can be used ex vivo to trigger a strong protection of cytotoxic lymphocytes against oxidative stress. This finding could have far reaching importance for the further improvement of cell products in treatment of cancer patients using ACT. Thus, we conclude that ex vivo AUF pretreatment of human NK-, CAR T cells and TIL is a new innovation that should be possible to apply in any existing expansion protocols used to generate ACT cell products for potentially improved clinical efficacy against hematological and solid tumors.

## Data Availability

Data are available on reasonable request. All data relevant to the study are included in the article or uploaded as online supplemental information.
