# Supplementary material for: Targeting of Nrf2 improves antitumoral responses by human NK cells, TIL and CAR T cells during oxidative stress
Source: J Immunother Cancer. 2022 Jun 22;10(6):e004458. doi: 10.1136/jitc-2021-004458 (PMC9226989; doi:10.1136/jitc-2021-004458)
Supplement: Supplementary data [file jitc-2021-004458supp001.pdf]

Supplementary information Renken et al.

Targeting of Nrf2 improves anti-tumoral responses by human NK cells, TIL and CAR T cells during oxidative stress

Content:

- Supplementary Materials and methods
- Figures
- Table

Supplementary Materials and methods:

Cells

K562, RAJI, N6/ADR and THP-1 were cultured in RPMI with 10 % FBS (LifeTechnologies). KASUMI, ANRU and BEHA tumor cell lines in IMDM (LifeTechnologies) 20 % FBS. EBV-LCL feeder cells (*1*) and KADA tumor cell line in RPMI 20 % FBS. ANRU, KADA and BEHA, were generated as previously described (*2*). Cells grown in suspension (K562, RAJI, N6/ADR, KASUMI-1, THP-1, LCL) were cultured at $0,5x10^6$ cells/ mL, while adherent cells (KADA, ANRU, BEHA) were passaged every 2-5 days using 0,05 % Trypsin- EDTA (LifeTechnologies). For generation of autologous tumor spheroids, 5000 ANRU tumor cells were seeded per well in Ultra-Low Attachment 96-well plates (Corning Costar) in culture medium containing 2 % Matrigel (Corning) for 3 days. Spheroids were used to analyse TIL mediated killing and TIL infiltration using confocal microscopy and flow cytometry, for details see each section.

For healthy donor NK cell expansion, a EBV-LCL feeder cell line was used, irradiated at 100 gy and then co- cultured with NK cells, at the ratio10:1 LCL:NK, in X-Vivo 20 (Lonza) supplemented with 10% human AB serum (Karolinska University Hospital) and 1000U/mL (from day three 500 U/mL) IL-2 (Proleukin, Novartis). From day 6 or 10, NK cells were kept at $0,5x10^6$ cells/ mL or 1 $x10^6$/mL, respectively. Purity of expanded NK cell was assessed at day 10 by flow cytometry, see below. All primary cells and cell lines were cultured at 37°C and 5% CO_2.

Supplementary information Renken et al.

Melanoma patient derived TIL and CD19 directed CAR T cells were generated as previously described (*3, 4*).

Co-culture with autologous monocytes

For monocyte experiments, autologous NK cell- monocyte or TIL- monocyte pairs were used. NK cells/TIL were isolated and pre-treated with 0,5 µg/mL AUF, as described above, and co-cultured with unstimulated or stimulated monocytes at indicated ratios. Monocytes were stimulated with 100 ng/ mL PMA (Sigma-Aldrich) for 1 min, washed and added at indicated ratio while the number of NK cells/ TIL was kept constant. As control, H_2O_2 was used at indicated concentration. Cells were co-cultured for 2h followed by staining for intracellular ROS (see flow cytometry) or ^{51}Cr release assay (effector; target ratio, E:T ratio 10:1). For NK cell experiments, non-expanded NK cells were used hence referred to as naïve.

Detection of ROS production by monocytes

Monocytes were stimulated with 100 ng/ mL PMA for 1 min and washed with HBSS (Gibco). Cells were resuspended in HBSS 5% FBS and added to a 96-well Optiplate (Perkin Elmer) containing HBSS 5% FBS and Luminol (final concentration 56 µM; Sigma-Aldrich). Luminescence was measured immediately using an EnSpire plate reader (Perkin Elmer).

Flow cytometry

For phenotypic analysis of NK cells (cell surface and intracellular markers), NK cells were AUF pre-treated as described above. After H_2O_2 treatment, NK cells were incubated for 2h in RPMI 2% AB serum and stained with respective antibodies. For detection of intracellular ROS levels, CellROX™ Deep Red Reagent (Invitrogen) was used. Briefly, NK cells were stained with 2,5 µM CellROX solution in RPMI for 30 min at 37°C and stained for flow cytometry with anti-CD56 and anti-CD3. TIL were pre-incubated with 10 µM CM-H2DCFDA (Thermo Fisher Scientific) solution in RPMI for 30 min at 37 °C prior to H_2O_2 treatment and then stained with anti-CD3, anti-CD4 and anti-CD8.For viability analysis, NK cells were washed after H_2O_2 treatment, cultured for 4h in RPMI 2% hAB serum and stained with Annexin V-FITC (Miltenyi Biotech) and propidium iodide (BD Biosciences). Viability of ANRU

TIL was assessed using Aqua Dead Cell Stain Kit (see above). To study TIL infiltration into spheroids, eight spheroids of each condition were pooled and carefully washed twice with PBS, dissociated with trypsin and stained with anti-CD3, anti-CD4 and anti-CD8. The supernatant was collected and stained to quantify the non-infiltrated fraction. Purity of expanded NK cell was determined at day 10 staining with anti-CD56, anti-CD3 and anti-CD19. To detect Tregs, TIL were treated with 0,5 µg/mL AUF as described and stained for CD3, CD4 and FoxP3 immediately or 72h after treatment using BD Transcription Factor Buffer Set following the manufacturer's instructions (BD Biosciences). For intracellular cytokine staining, KADA TIL were co-cultured with autologous tumor cells (E:T 4:1) or cultured with 25 ng/mL PMA (Sigma Aldrich) and 500 ng/mL Ionomycin (Sigma Aldrich) for 6h. After 2h, GolgiStop™ and GolgiPlug™ (BD Bioscience) were added. Cells were then stained for CD3, CD4, CD8, IL-10 and TGFβ as described.

Confocal microscopy

After TIL-spheroid co-culture, spheroids were washed, fixed with 4% PFA (Thermo Scientific) and stained with anti-CD8a followed by the secondary antibody (goat-anti-mouse IgG-AF647) and Hoechst 33342 dye (Invitrogen). Spheroids were cleared with 88% glycerol (Sigma) overnight, transferred to 8-well µ-slides (Ibidi) and imaged with the Zeiss LSM800 confocal microscope. Quantification of CD8+ T cells was done using QuPath(*5*).

Evaluation of Nrf2 target gene expression

Lymphocytes were pre-treated with AUF, SUL or DMF as described above. RNA was isolated using TRIzol™ Plus RNA Purification Kit (Invitrogen). cDNA was generated using the iScript™ cDNA Synthesis Kit (Bio-Rad) and qPCR was done using iTaq™ Universal SYBR® Green Supermix (Bio-Rad) in the CFX96 Touch Real-Time PCR Detection System (Bio-Rad). Fold change expression from untreated cells was calculated using the 2^-ΔΔCt formula with b-actin as reference gene. Evaluated genes were NAD(P)H Quinone Dehydrogenase 1 (NQO1), Kelch Like ECH Associated Protein 1 (Keap1), Heme Oxygenase 1 (HMOX1) and Thioredoxin Reductase 1 (TXNRD1). For primer sequences (5'-3') see Table S1.

Supplementary information Renken et al.

Reference Supplementary Material and Methods

1. A. Lundqvist, M. Berg, A. Smith, R. W. Childs, Bortezomib Treatment to Potentiate the Anti-tumor Immunity of Ex-vivo Expanded Adoptively Infused Autologous Natural Killer Cells. *J Cancer* **2**, 383-385 (2011).
2. S. L. Wickström, T. Lövgren, M. Volkmar, B. Reinhold, J. S. Duke-Cohan, L. Hartmann, J. Rebmann, A. Mueller, J. Melief, R. Maas, M. Ligtenberg, J. Hansson, R. Offringa, B. Seliger, I. Poschke, E. L. Reinherz, R. Kiessling, Cancer Neoepitopes for Immunotherapy: Discordance Between Tumor-Infiltrating T Cell Reactivity and Tumor MHC Peptidome Display. *Front Immunol* **10**, 2766 (2019).
3. T. Lövgren, M. Wolodarski, S. Wickström, U. Edbäck, M. Wallin, E. Martell, K. Markland, P. Blomberg, M. Nyström, A. Lundqvist, H. Jacobsson, G. Ullenhag, P. Ljungman, J. Hansson, G. Masucci, R. Tell, I. Poschke, L. Adamson, J. Mattsson, R. Kiessling, Complete and long-lasting clinical responses in immune checkpoint inhibitor-resistant, metastasized melanoma treated with adoptive T cell transfer combined with DC vaccination. *Oncoimmunology* **9**, 1792058 (2020).
4. I. Magalhaes, I. Kalland, J. N. Kochenderfer, A. Österborg, M. Uhlin, J. Mattsson, CD19 Chimeric Antigen Receptor T Cells From Patients With Chronic Lymphocytic Leukemia Display an Elevated IFN-γ Production Profile. *J Immunother* **41**, 73-83 (2018).
5. P. Bankhead, M. B. Loughrey, J. A. Fernández, Y. Dombrowski, D. G. McArt, P. D. Dunne, S. McQuaid, R. T. Gray, L. J. Murray, H. G. Coleman, J. A. James, M. Salto-Tellez, P. W. Hamilton, QuPath: Open source software for digital pathology image analysis. *Scientific Reports* **7**, 16878 (2017).

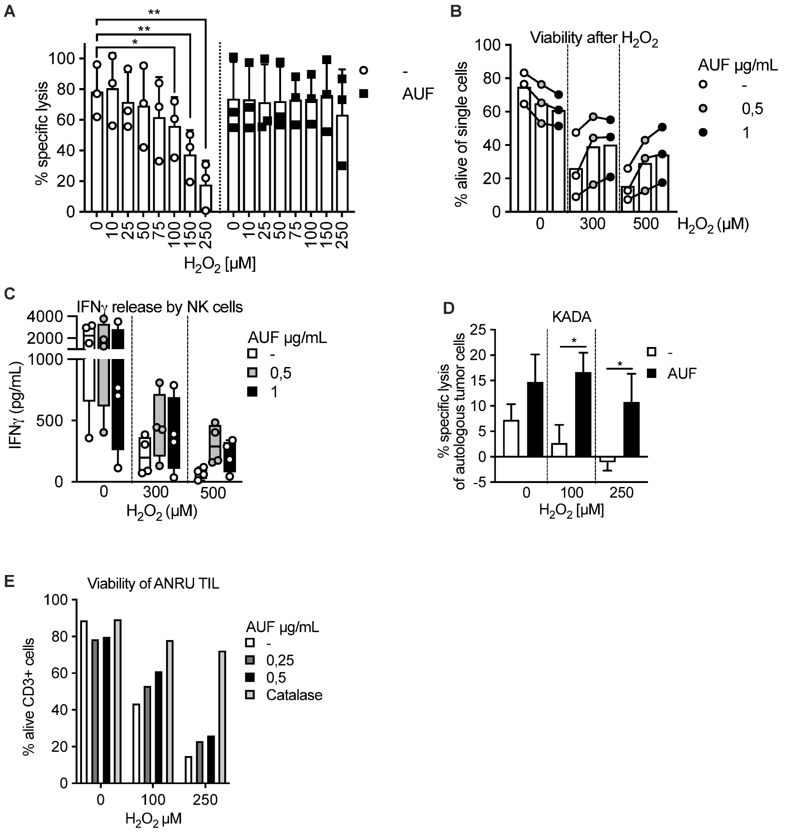

Suppl. Figure 1.

a Lysis of K562 cells (E:T 9:1) by NK cells after H_2O_2 treatment, n=3, paired t-test. **b** Frequency of alive NK cells (% of single cells) 4h after H_2O_2 treatment, n=3. **c** IFNγ release by AUF pre-treated NK cells during co-culture with K562 cells, n=4. **d** Lysis of KADA tumor cells by autologous, AUF pre-treated TIL after H_2O_2 treatment, n=3, unpaired t-test. **e** Viability of ANRU TIL after H_2O_2 treatment and subsequent co-culture with autologous tumor cells for 24h. ***p<0.001, **p<0.01, *p<0.05.

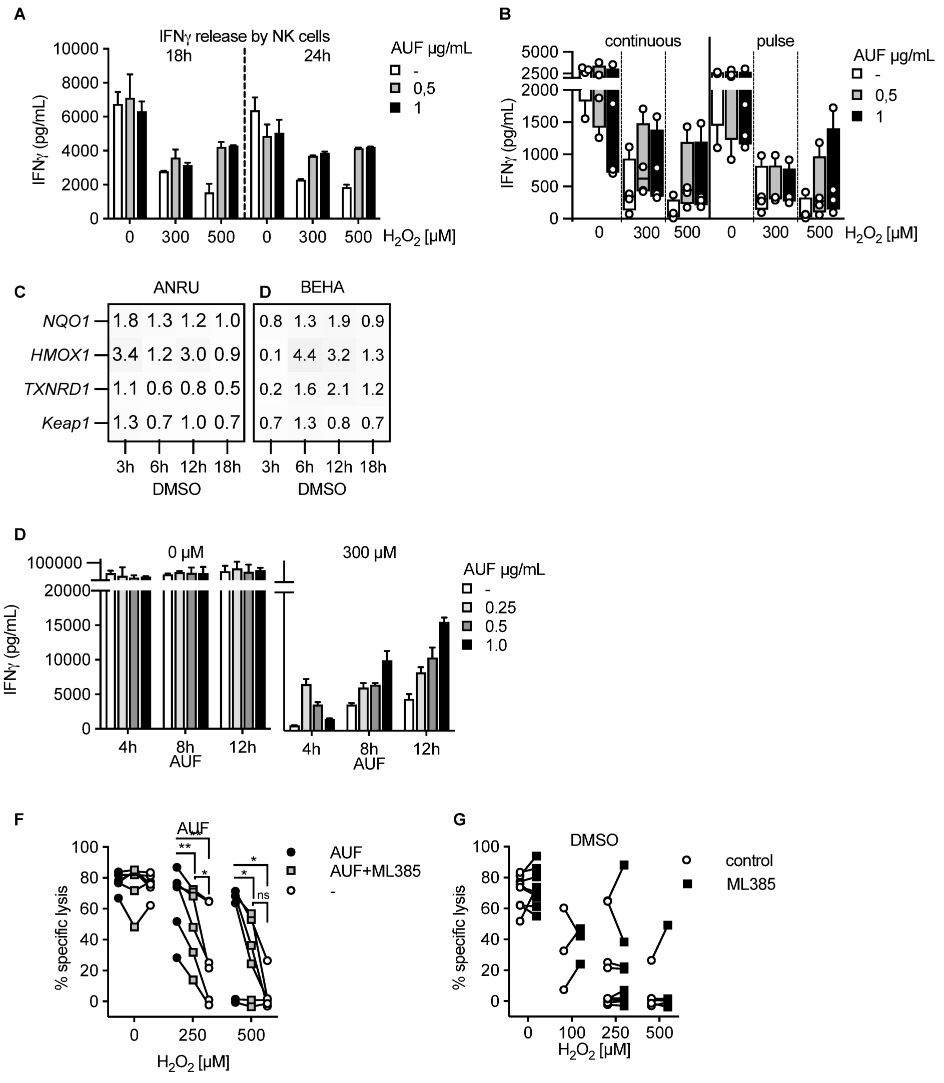

Suppl. Figure 2

A NK cells were pre-treated for 18h or 24h with AUF, treated with H_2O_2 and then co-cultured with K562 cells, n=1. **B** IFNγ release by NK cells pre-treated with AUF either for 18h continuously or pulse treated for 30 min followed by 17,5h culture without AUF, n=4. Overlapping datapoints with Suppl. Figure 1B. **C-D** Quantification of Nrf2 target gene expression in TIL treated with DMSO for indicated durations. C, ANRU and D, BEHA. **D** IFNγ release by expanded, AUF pre-treated $CD8^+$ T cells after H_2O_2 treatment and CD3/CD28 bead stimulation. **E** Lysis of K562 by NK cells pre-treated with either DMSO, AUF or a combination of AUF and ML385. **F** Lysis of K562 cells by NK cells pre-treated with DMSO +/- the Nrf2 inhibitor ML385. E-F, overlapping datapoints with Figure 2G.

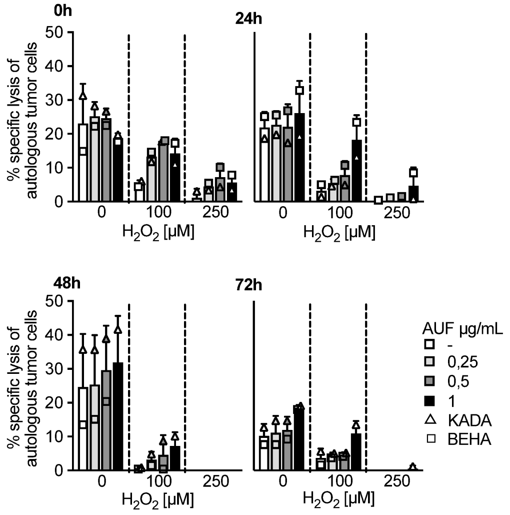

Suppl. Figure 3
Lysis of autologous tumor cells by KADA and BEHA TIL. TIL were pre-treated with AUF for 18h and then cultured without AUF for indicated timepoints before H_2O_2 treatment and co-culture. n=2.

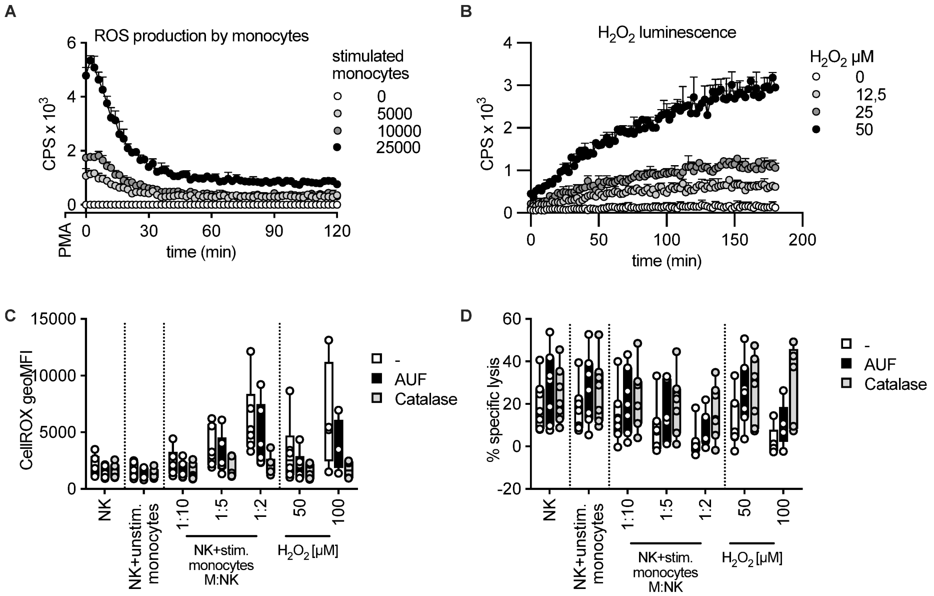

Suppl. Figure 4.

A-B Representative plots showing luminol-based detection of ROS, measured as luminescence (counts per seconds, CPS). **A** Cell-number dependent ROS production by monocytes. **B** Detection of H_2O_2. **C-D A**uranofin (0,5 µg/mL) pre-treated NK cells or control NK cells with or without the addition of catalase were co-cultured with autologous monocytes for two hours and then tested for **C** intracellular ROS levels, n=7. or **D** lysis of K562 cells, n=7. Data for control NK and AUF treated NK in C and D are also presented in figure 4 C-D. One data point represents one NK cell donor.

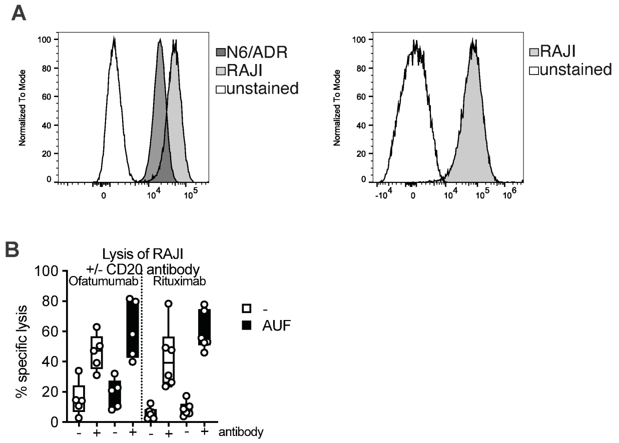

Suppl. Figure 5
A Characterization of CD19 and CD20 expression in RAJI and N6/ADR cell lines using flow cytometry. **B** Lysis of RAJI cells (E:T 9:1) by NK cells in the presence (+) or absence (-) of Ofatumumab (n=5) or Rituximab (n=6). Overlapping data points with Figure 5A

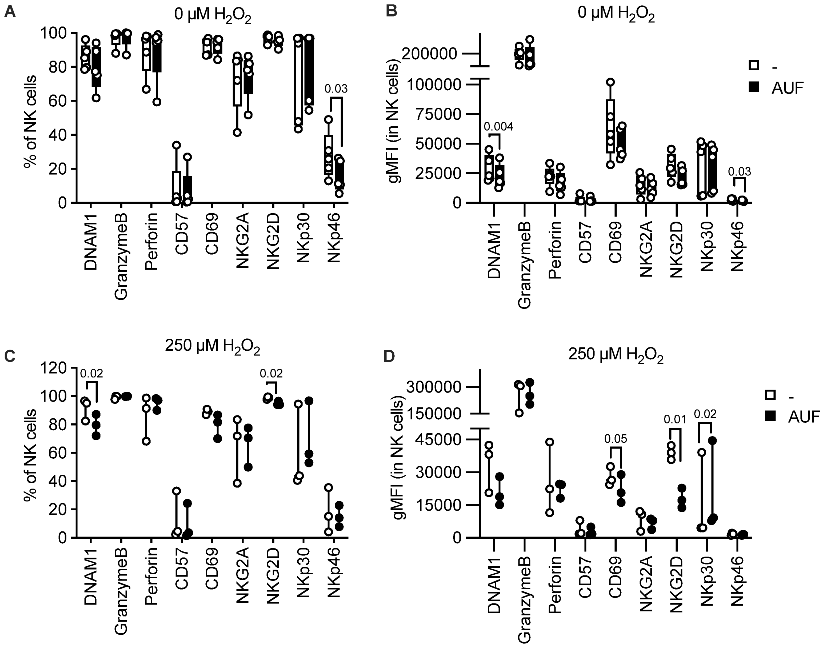

Suppl. Figure. 6
NK cells were pre-treated with AUF, exposed to 0 µM (**A-B**) or 250 µM **(C-D)** H_2O_2, cultured for 2h and then stained for flow cytometry. Presented is the frequency of alive NK cells positive for the respective marker (A, C) or the geometric MFI (B,D). N= 3-4.

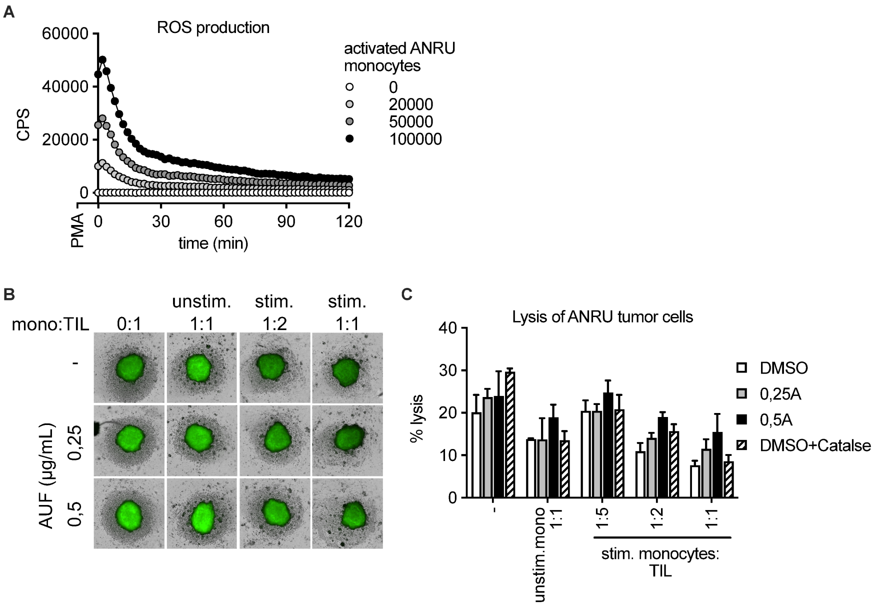

Suppl. Figure 7

A Luminol-based detection of ROS produced by PMA stimulated ANRU monocytes. **B** Representative live cell images showing caspase 3/7 activation in ANRU spheroids after 48h co-culture with autologous TIL. TIL were cultured with monocytes prior to being added to the spheroid. **C** Lysis of ANRU tumor cells by autologous TIL that had been pre-cultured with monocytes. Representative data from one out of three experiments.

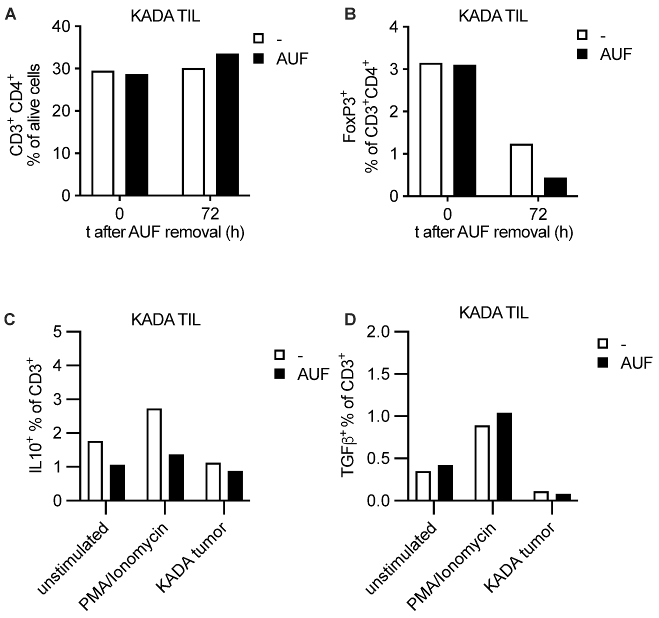

Suppl. Figure 8
KADA TIL were treated with AUF (0,5 µg/mL), washed and cultured for indicated durations (t, hours) before quantification of CD4+ T cells (**A**) and Tregs (**B**). Tregs were defined as $CD3^{+}$ $CD4^{+}$ $FoxP3^{+}$. Directly after the treatment, TIL were stimulated for six hours with PMA/Ionomycin or autologous tumor cells and then stained for intracellular cytokines IL-10 (**C**) and TGFβ (**D**).

Supplementary Tables

REAGENT or RESOURCE	SOURCE	IDENTIFIER
Antibodies		
PE/Cyanine7 anti-human CD56 (NCAM) (clone HCD56)	BioLegend	Cat# 318318 RRID:AB_604107
Pacific Blue anti-human CD3 antibody (clone UCHT1)	BioLegend	Cat# 300431 RRID:AB_1595437
PerCP/Cyanine5.5 anti-human CD3 antibody (clone SK7)	BioLegend	Cat# 344808 RRID:AB_10640736
FITC anti-human CD19 antibody (clone SJ25C1)	BioLegend	Cat# 363008 RRID:AB_2564171
Brilliant Violet 570 anti-human CD19 antibody (clone HIB19)	BioLegend	Cat# 302235 RRID:AB_10901168
APC/Cyanine7 anti-human CD20 antibody (clone 2H7)	BioLegend	Cat# 302313 RRID:AB_314261
Pacific Blue anti-human CD16 antibody (clone 3G8)	BD Biosciences	Cat# 558122 RRID:AB_397042
FITC anti-human CD107a (LAMP-1) antibody (clone H4A3)	BioLegend	Cat# 328606 RRID:AB_1186036
PE anti-human IFNγ antibody (clone B27)	BioLegend	Cat# 506506 RRID:AB_ 315440
PerCP anti-human CD4 (clone OKT4)	BioLegend	Cat# 317432 RRID: AB_2028494
APC-Cyanine7 anti-human CD8 (clone Sk1)	BioLegend	Cat# 344714 RRID: AB_2044006
Purified anti-human CD8a antibody (clone CD8/144B)	BioLegend	Cat# 372902 RRID:AB_2650657
Alexa Fluor 647 Goat anti-mouse IgG (minimal x-reactivity) antibody (clone Poly4053)	BioLegend	Cat# 405322 RRID:AB_2563045
Brilliant Violet 785 anti-human CD226 (DNAM-1) antibody (clone 11A8)	BioLegend	Cat# 338321 RRID:AB_2721559
APC anti-human/mouse Granzyme B Recombinant antibody (clone QA16A02)	BioLegend	Cat# 372203 RRID:AB_2687027
FITC anti-human Perforin antibody (clone B-D48)	BioLegend	Cat# 353309 RRID:AB_2571966
Brilliant Violet 605 anti-human CD57 Recombinant antibody (clone QA17A04)	BioLegend	Cat# 393303 RRID:AB_2728425

APC/Cyanine7 anti-human CD69 antibody (clone FN50)	BioLegend	Cat# 310914<br>RRID:AB_314849
PE anti-human CD159a (NKG2A) antibody (clone S19004C)	BioLegend	Cat # 375103<br>RRID:AB_2888861
PE-CF594 Mouse Anti-Human CD314 (NKG2D) (clone (1D11)	BD Biosciences	Cat# 562498<br>RRID:AB_11151913
Alexa Fluor® 647 Mouse anti-Human CD337 (NKp30) (clone p30-15)	BD Biosciences	Cat# 558408<br>RRID:AB_647154
Alexa Fluor ® 488 anti-human CD335 (NKp46) antibody (clone 9E2)	BioLegend	Cat# 331937<br>RRID:AB_2715915
APC-Cy™7 Mouse Anti-Human CD3 (clone SK7)	BD Biosciences	Cat# 561800<br>RRID:AB_10895381
Alexa Fluor ® 488 Mouse Anti-Human TGF-β1 (clone TW4-9E7)	BD Biosciences	Cat# 562545<br>RRID: AB_2737645
Brilliant Violet 421 ™ anti-human IL-10 Antibody (clone JES3-9D7)	BioLegend	Cat#501421<br>RRID:AB_ 10896947
FITC anti human FOXP3 (clone PCH101)	eBioscience (Invitrogen)	Cat# 11-4776-42<br>RRID: AB_1724125
Biological samples		
Healthy donor peripheral blood samples (buffy coats) (NK cells and monocytes)	Karolinska University Hospital Blood Bank	N/A
CD19 CAR T cells	Generated by Isabelle Magalhaes, Jonas Mattsson Laboratory, Karolinska Institutet	N/A
Melanoma tumor tissue; autologous tumor TIL pairs	Kiessling laboratory; Stockholms medicinska biobank	N/A
Chemicals		
Catalase from bovine liver	Sigma-Aldrich	C1345<br>CAS: 9001-05-2
ML385	Sigma-Aldrich	SML1833<br>CAS: 846557-71-9
Auranofin	Sigma-Aldrich	A6733<br>CAS: 34031-32-8
DL- Sulforaphane	Sigma-Aldrich	S4441<br>CAS: 4478-93-7

Dimethyl Fumarate	Sigma-Aldrich	242926<br>CAS: 624-49-7
Hydrogen peroxide solution, 30%	Sigma-Aldrich	H1009<br>CAS: 7722-84-1
Rituximab (MabThera)	Roche	N/A
Ofatumumab (Arzerra)	Novartis	N/A
Luminol	Sigma-Aldrich	123072<br>CAS: 521-31-3
Critical commercial assays		
CellEvent™ Caspase-3/7 Green Detection Reagent	Invitrogen	Cat# C10423
CellROX™ Deep Red Reagent	Invitrogen	Cat# C10422
Human IFN-γ ELISA development kit	Mabtech	3420-1H-20
^{51}Cr release assay (Sodium Chromate and LumaPlates)	PerkinElmer	Cat# NEZ030 and 6006633
Experimental models: Cell lines		
K562	ATCC	CCL-243
KASUMI-1	ATCC	CRL-2724
THP-1	ATCC	TIB-202
RAJI	ATCC	CCL-86
EBV-LCL feeder cells		
N6/ADR	ATCC	CRL-3274
Primary melanoma cell lines: KADA, ANRU, BEHA	Kiessling laboratory, Karolinska Institutet	N/A
Oligonucleotides		
Primer Keap1 forward TGCGTCCTGCACAACTGTATC	This paper, ThermoFisher	N/A
Primer Keap1 reverse CCAGGAACGTGTGACCATCA	This paper, ThermoFisher	N/A
Primer NQO1 forward CTGAAGGACCCTGCGAACT	This paper, ThermoFisher	N/A
Primer NQO1 reverse TCGCTCAAACCAGCCTTTCAG	This paper, ThermoFisher	N/A
Primer HMOX1 forward ACTCCCTGGAGATGACTCCC	This paper, ThermoFisher	N/A

Primer HMOX1 reverse TCTTGCACTTTGTTGCTGGC	This paper, ThermoFisher	N/A
Primer TXNRD1 forward ATATGGCAAGAAGGTGATGGTCC	This paper, ThermoFisher	N/A
Primer TXNRD1 reverse GGGCTTGTCCTAACAAAGCTG	This paper, ThermoFisher	N/A
Primer b-actin forward CTCGCCTTTGCCGATCCG	This paper, ThermoFisher	N/A
Primer b-actin reverse TCTCCATGTCGTCCCAGTTG	This paper, ThermoFisher	N/A
Software and algorithms		
Graphpad Prism, version 9	Graphpad Software, San Diego, California USA,	www.graphpad.com
FlowJo™ Software, version 10	Becton, Dickinson and Company	https://www.flowjo.com/solutions/flowjo
Incucyte Base Software<br>Incucyte Spheroid Analysis Software Module	Essen Bioscience	N/A<br>Cat# 9600-0019
Zeiss ZEN lite 3.1 blue edition	Zeiss	https://www.zeiss.com/microscopy/int/products/microscope-software/zen-lite.html
QuPath image analysis software		https://qupath.readthedocs.io/en/stable/index.html#
Other		

Table S1: Detailed information about reagents, biological samples, and computer software.
